# HPV infection in ENT - certainties and assumptions

**DOI:** 10.1186/1471-2334-13-S1-P111

**Published:** 2013-12-16

**Authors:** Loredana Mitran, Mihai Mitran, Daniela Safta, Sorin Puia, Carmen Georgescu

**Affiliations:** 1 Elias University Emergency Hospital, Bucharest, Romania; 2 Clinical Hospital of Obstetrics and Gynecology “Prof. Dr. Panait Sârbu”, Bucharest, Romania

## Background

HPV infection is considered the most frequent sexually transmitted infection. The clinical manifestations of infection are various and variable, including the ones in ENT, such as recurrent laryngeal papillomatosis and tonsillar cancer.

## Case report

We present the possibilities of diagnosis of HPV infection in ENT, and the therapeutic, prophylactic and curative modalities, as well as the assumptions and prevention goals through vaccination within this specialty. We communicate the management of three clinical cases of laryngeal papillomatosis in adults.

The first case, a 75-year-old woman, 4 times under surgical intervention with “cold surgery”, vaccinated with Silgard. The second case, a 55-year-old woman, also 4 times under surgical intervention receives Gardasil after the last intervention. The third case, a 35-year-old man, 2 times surgically operated after diagnosed with papiloma scuamosis and vaccined with Silgard.

All the above mentioned cases don’t present any regression of the disease during the last three years.

## Conclusion

The involvement of HPV in ENT cancer pathology is undeniable. Laryngeal papillomatosis is a precancerous condition caused by HPV strains 6 and 11. HPV vaccination can be useful both to prevent HPV-induced pathology in otolaryngology and after surgery, to prevent recurrence.

